# Bacterial Endophytes Contribute to Rice Seedling Establishment Under Submergence

**DOI:** 10.3389/fpls.2022.908349

**Published:** 2022-06-29

**Authors:** Germán Darío Ahumada, Eva María Gómez-Álvarez, Matteo Dell’Acqua, Iris Bertani, Vittorio Venturi, Pierdomenico Perata, Chiara Pucciariello

**Affiliations:** ^1^Center of Plant Sciences, Scuola Superiore Sant’Anna, Pisa, Italy; ^2^International Centre for Genetic Engineering and Biotechnology, Trieste, Italy

**Keywords:** coleoptile, microbiota, *Oryza sativa*, hypoxia, rice

## Abstract

Flooding events caused by severe rains and poor soil drainage can interfere with plant germination and seedling establishment. Rice is one of the cereal crops that has unique germination strategies under flooding. One of these strategies is based on the fast coleoptile elongation in order to reach the water surface and re-establish the contact with the air. Microorganisms can contribute to plant health via plant growth promoters and provide protection from abiotic stresses. To characterise the community composition of the microbiome in rice germination under submergence, a 16S rRNA gene profiling metagenomic analysis was performed of temperate *japonica* rice varieties Arborio and Lamone seedlings, which showed contrasting responses in terms of coleoptile length when submerged. This analysis showed a distinct microbiota composition of Arborio seeds under submergence, which are characterised by the development of a long coleoptile. To examine the potential function of microbial communities under submergence, culturable bacteria were isolated, identified and tested for plant growth-promoting activities. A subgroup of isolated bacteria showed the capacity to hydrolyse starch and produce indole-related compounds under hypoxia. Selected bacteria were inoculated in seeds to evaluate their effect on rice under submergence, showing a response that is dependent on the rice genotype. Our findings suggest that endophytic bacteria possess plant growth-promoting activities that can substantially contribute to rice seedling establishment under submergence.

## Introduction

Severe rains at unexpected periods of the season and in geographical areas which are not prone to excessive rainy events, coupled with a low capacity of the soil to drain water, expose plants to the risk of submergence during germination and seedling establishment. However, rice can germinate successfully even in anoxic environment (Kordan, 1972; Alpi and Beevers, 1983; Perata and Alpi, 1993), which is a unique ability that is the consequence of complex physiological and molecular mechanisms. These includes the ability to express α-amylase enzymes in the seeds to hydrolyse starch, and to mobilise sugars from the endosperm to sink organs (Perata et al., 1992; Guglielminetti et al., 1995; Kretzschmar et al., 2015).

The germination and seedling development of rice under O_2_ shortage is initially characterised by the emergence of the coleoptile, while the radicle growth is dampened (Narsai et al., 2015). In anoxia-tolerant rice genotypes, the coleoptile elongates rapidly and acts like a “snorkel” in order to supply O_2_ to the submerged tissues (Kordan, 1974). Efficient coleoptile elongation under submergence has been associated with the presence and transport of auxin in *japonica* rice (Nghi et al., 2021). In air, the radicle protrudes while the coleoptile rapidly splits and senescence sets in for the extension of true leaves (for a review see Pucciariello, 2020).

A plant’s performance is also associated with its microbiota, which contributes to plant health (Hardoim et al., 2015) protecting the host from pathogens, promoting plant growth and increasing abiotic stress resilience (Sessitsch et al., 2019). Notably, endophytic bacteria support the growth of cactus seeds in desert areas, contributing to rock weathering and soil formation (Puente et al., 2009). Endophytic bacteria isolated from desert plants were shown to promote plants growth of maize plants (ALKahtani et al., 2010), while bacterial endophytes isolated from native halophytic plants mitigate the effect of salt stress on bean plants (Mahgoub et al., 2021). This suggests that under extreme environmental conditions, such as submergence, bacteria may be crucial to plant development and have a strong potential for application in agriculture (Eid et al., 2021).

Bacteria can promote plant growth through direct or indirect mechanisms. Plant growth promoting bacteria (PGPB) can produce phytohormones such as auxins, which were found to be involved in coleoptile elongation under submergence (Nghi et al., 2021). Other microbial functions include phosphate solubilisation, nitrogen fixation, and the production of siderophores (Compant et al., 2019). Bacteria can also impede growth and the activity of pathogenic microorganisms via competition for space and nutrients, antibiosis, inhibition of toxins, and induction of plant defence mechanisms (Weyens et al., 2009).

In seeds, bacteria can be found in the outer and inner seed coats, as well as in the embryonic tissues (Rodríguez et al., 2018). During seed maturation, the accumulation of starch and the loss of water favour endophytes that are able to tolerate high osmotic pressure and produce endospores (Mano et al., 2006). These microbes can survive desiccation and seed storage conditions (Truyens et al., 2015), and often possess amylolytic activity, enabling them to degrade starch (Mano et al., 2006).

Among endophytes, microbes residing in seeds are inherited by the next generation, giving rise to a vertical transmission that influences the offspring in the initial phases of life (Mitter et al., 2017). In fact, as primary colonisers of the host, seed endophytes can affect germination, seedling establishment, and plant survival (Rodríguez et al., 2018). In some cases, existing microbes in the plant seeds form the basis for a successful plant establishment before the arrival of the soil microbes. Certain microbes are likely to have evolved in co-selection with plants, thus contributing to different traits and finally to the *holobiont* evolution in the environment (Hardoim et al., 2012). Very interestingly, establishment of the seed microbiota is believed to be the result of specific interactions between plant and bacterial genotypes (Rodríguez et al., 2018).

In this study, we analysed Arborio and Lamone *japonica* rice varieties, showing contrasting phenotype when germinating under submergence, using metagenomics, *in vivo* bacterial isolation, and PGP assays. Our findings suggest that seed endophytic bacteria possess PGP activities, i.e., amylase activity and capacity to synthesise indole-related compounds, that are functional also under hypoxia and that can positively contribute to rice seedling establishment under submergence.

## Materials and Methods

### Plant Materials

Rice (*Oryza sativa L*.) genotypes Arborio, Lamone, Arsenal and Ermes seeds were sourced from the Rice Germplasm Collection of the CREA-Research Centre for Cereal and Industrial Crops (Vercelli, Italy). Arborio and Ermes are characterised by a long coleoptile under submergence, while Lamone and Arsenal harbour a short coleoptile (Nghi et al., 2019, 2021). Seeds were taken from rice grown in a paddy field under an irrigated cropping system with standard crop management (coordinates 45°19’24.00” N; 8°22’26.28” E; 134 m.a.s.l.), characterised by a sandy type soil (sand 47.8%, loam 42.8%, clay 9.41%) with pH 6.36 (Volante et al., 2017).

Seeds were stored until use at 4°C in a controlled refrigerator (LAB-MIDI, Desmon Scientific, Nusco, Italy). Rice seeds were de-hulled and surface sterilised following the protocol of Bertani et al. (2016). Briefly, seeds were treated with 75% ethanol for 2 min, 3.5% sodium hypochlorite for 2 min, and finally 75% ethanol for 1 min. Seeds were washed with sterile water at each step, and several times at the end of the process. To verify the efficiency of the standard sterilisation, 1 mL of the final washing solution and five sterilised rice seeds were incubated on Luria Bertani (LB) culture media at RT under air and hypoxia. The plates were checked for bacterial growth for the following 72 h. Once the process of standard sterilisation was successful, seeds were used for further analyses.

### Submergence Experiment and Sampling

Sterilised seeds were germinated in Magenta GA-7 vessels. Seeds in air were germinated in vessels on sterilised wet filter paper. Seeds under submergence were germinated fulfilling the vessel to the top with 360 mL sterile water. The experiments were performed in triplicates for each variety under air and submergence, using 10 to 15 seeds for each replicate, in a growing cabinet (Percival Scientific, Perry, Iowa, United States) at 30°C and 57% RH in the dark for four days. For the culturable bacteria isolation, at day 4, a total of 1 g of entire rice seedlings from the bulk tissue of each replicate was ground using a sterile mortar and pestle in 1 mL of phosphate buffer saline (PBS, pH 7), and subsequently stored at −80°C in 20% glycerol. For metagenomics analysis, at day 4, a total of 1 g of rice entire seedlings from bulk tissue of each replicate was stored at −80°C and subsequently sent to IGATech Technology Services (Udine, Italy) for DNA extraction and further analysis. The absence of external contamination in these steps was checked by incubating the submergence water and seedlings as described above.

### Metagenomics and Statistical Data Analysis

DNA was extracted by bulk tissue of day 4 rice Arborio and Lamone seedlings using the IGATech standard protocol of the Dneasy mericon Food kit (Qiagen, Venlo, NL). The hypervariable V3 and V4 regions of the 16S rRNA gene were amplified using the 16S-341F 5′-CCTACGGGNGGCWGCAG-3′ and 16S-805R 5′-GACTACHVGGGTATCTAAT-3′ primer sets (Klindworth et al., 2013). An initial PCR amplification was performed using locus specific PCR primers and applying PNA clamping to block amplification of host chloroplast and mitochondrial sequences, following the manufacturer’s protocol (PNA Bio Inc, Newbury Park, CA, United States). A subsequent amplification integrated relevant flow-cell binding domains and unique indices (NexteraXT Index Kit, Illumina, San Diego, CA, United States). Libraries were sequenced on an MiSeq instrument (Illumina, San Diego, CA, United States) using the 300 bp paired-end mode.

The quality of the sequencing process was checked using FastQC (Andrews, 2010). Low quality paired-end Illumina reads were removed using ERNE-FILTERING software (Del Fabbro et al., 2013), applying a minimum PHRED score (-q) of 20.

The resulting sequences were imported into the QIIME2 platform (Caporaso et al., 2010) (v. 1.9.0). After the denoising step using the DADA2 plugin of the QIIME2 pipeline, high quality sequences were converted into operational taxonomic units (OTUs). The resulting OTU table was filtered to remove chimeras, but to retain borderline entries, using the UCHIME plugin of the QIIME2 pipeline. OTU representative sequences were matched to the SILVA (Ribosomal RNA Gene Database Project Data^1^) reference database (Quast et al., 2013) (v. 132), with 99% sequence identity. The OTU table and the taxonomy matrix produced were used for further analysis.

Statistical analyses were performed using R (R Core Team, 2021). A phyloseq object was created using R/Phyloseq (McMurdie and Holmes, 2013). Reads belonging to mitochondria, chloroplasts and unassigned features were removed before further analysis.

To proceed with the Alpha-diversity analysis, the OTU table was rarefied to the minimum library size. The Chao1 and Shannon indexes were calculated using the “estimate_richness” function of R/Phyloseq. After the Saphiro-Wilk test, a Kruskal-Wallis Rank Sum non-parametric analysis of variance was performed on the four sample types, followed by the Wilcoxon Rank Sum test for variety and condition variables.

Data were then normalised by cumulative sum scaling (CSS) using cumNorm() from the R/MetagenomeSeq v.3.8 (Paulson et al., 2013). Beta-diversity was analysed using a canonical analysis of principal coordinates (CAP) of the ordinate() function of the R/Phyloseq. Beta diversity was studied in non-rarefied, normalised data. Differences were computed using Bray-Curtis and weighted Unifrac distances, which were confirmed by permutational multivariate analysis of variance (PERMANOVA) for both distance matrixes. Samples were clustered using R/MClust and R/Cluster (Scrucca et al., 2016) (Supplementary Figure 1).

Low abundance OTUs and non-prevalent phyla were removed when they appeared less than 15 times in three replicates of the same group of samples. Relative abundance was analysed using the plot_bar function of R/Phyloseq. For the family barplot, only the 20 most abundant families were considered.

Differential abundance analysis to identify individual significant genus was performed with the R/MetagenomeSeq, using the zero-inflated Gaussian model with FDR calculation. Data were transformed to log(1 + x). GGplot2 package (Wickham, 2016) was used to visualise the data for boxplots.

Functional prediction of 16S rRNA gene amplicon data was performed using MicFunPred tool (Mongad et al., 2021) using the OTU abundance table. The analysis was conducted using Pfam database (Li et al., 2014; Keller-Costa et al., 2021; Mistry et al., 2021). Pfam results were analysed using R (R Core Team, 2021). ANOVA analysis (p-value < 0.001) corrected by Benjamini-Hochberg method was used to detect significant differences among Pfam groups represented in plant-related bacteria. Pfam groups were manually assigned to GO molecular function. Pheatmap() function from phetaman package (Kolde, 2012) was used to visualise the results of MicFunPred.

### Bacteria Isolation and Identification

Arborio and Lamone rice seedling homogenates were threefold diluted with PBS and plated in triplicate on four different culture media: 1/10 Tryptic Soy Agar (TSA, Difco BD, Franklin Lakes, New Jersey, United States), 1/5 Nutrient Broth (NB), LB and Reasoner’s 2A (R2A) 1.6% agar medium. Different isolation media were used in order to widening the panorama of diversity among isolated bacteria. Rice homogenates were subsequently incubated on TSA, NB, LB and R2A plates and grown under dark aerobic conditions at 30°C for 48 h (Moronta-Barrios et al., 2018). Rice homogenates were also incubated on TSA, NB, LB and R2A plates and grown under dark hypoxic conditions at 30°C for up to 10 days using anaerobic jars (Schuett Biotec GmbH, Gottingen, Germany). The anaerobic jars were filled with high-purity N_2_ and controlled using Oxoid anaerobic indicators (Thermo Fisher Scientific, Waltham, MA, United States).

Independent bacterial colonies were harvested and suspended in 100 μl of sterile H_2_O. Colony PCR was performed after heating up suspensions at 95°C for 5 min and centrifugation at 13,000 rpm for 10 min (Bertani et al., 2016; Moronta-Barrios et al., 2018; Bez et al., 2021). The full length of the 16S RNA gene was amplified by polymerase chain reaction (PCR), using 27F and 1492R primer sets (Edwards et al., 1989). For *Pantoea ananatis*, used for plant inoculum, a short length of the *GyrA* and *RecA* genes was also amplified (Das et al., 2014). PCR amplification was performed in 20 μl reaction containing 100 ng of genomic DNA and 0.4 U of Phusion High Fidelity DNA Polymerase (Thermo Fisher Scientific, Waltham, MA, United States), following standard procedures. PCR products were purified using Wizard^®^ SV Gel and PCR Clean-up System (Promega, Madison, WI, United States) for sequencing analysis. The sequences found were analysed using SILVA and NCBI^2^ databases. Some isolates were eliminated when they represented known human opportunistic species.

### Testing of *in vitro* Plant Growth Promotion Traits

Putative bacterial endophytes were tested for PGP activities in aerobic conditions and hypoxia at 30°C for 2 days (the conditions used for rice growth). PGP activities under hypoxic conditions were performed using a plexiglas chamber purged with high-purity N_2_ to provide a hypoxic environment. The O_2_ concentration (0.2 - 0.3%) was measured using a contactless O_2_ sensor FireStingO_2_ high-precision, PC-controlled fibre-optic O_2_ metre together with OXSP5 sensor spots (Pyro Science, Aachen, Germany). Plates were then incubated in anaerobic jars, as described above.

A starch hydrolysis *in vitro* assay was carried out on starch agar medium with the addition of Lugol staining solution containing 0.33% I_2_ and 0.67% KI (Zimbro et al., 2009).

An *in vitro* assay for indole related compounds, including indole-3-acetic acid (IAA), was performed using TSA 0.06% (w/v) medium containing 5 mM L-tryptophan and covered with a nitrocellulose membrane. After colony growth, the membrane was placed onto Whatman paper saturated with Salkowsky reagent (FeCl_3_ 1 mM; 35% perchloric acid), according to Bric et al. (1991).

*In vitro* N_2_ fixation capacity was tested on minimal M_9_ medium comparing bacteria growth with and without the N source (Penrose and Glick, 2003). The N_2_ fixation capacity was not tested in hypoxic conditions, since the low O_2_ environment was obtained with high purity N_2_ flux.

The solubilisation of phosphate by bacteria isolates was tested on the National Botanical Research Institute’s Phosphate (NBRIP) medium and was followed by visual observation (Nautiyal, 1999). Phosphate solubilisation capacity was not tested in hypoxic conditions, since the method of analysis needs O_2_ to work (Zeng et al., 2017).

*In vitro* activities were repeated three times for each isolate with similar results.

### Antibiotic Susceptibility Test and Bacteria Inoculum

A bacteria antibiotic susceptibility test was performed using the EUCAST disk diffusion method (v. 8.0^3^) with a few modifications. A total of 100 μl of inoculum (OD_600_ of 0.1) grown at 30°C was spread on LB agar plates. Oxoid™ (ThermoFisher Scientific, Waltham, MA, United States) antimicrobial susceptibility discs for cefotaxime (30 μg), gentamicin (30 μg), penicillin (6 μg), tetracycline (30 μg), ampicillin (10 μg), kanamycin (30 μg), cefazolin (30 μg) and streptomycin (25 μg) were placed on bacteria-inoculated LB agar plates. The inhibition area was measured using a Lyon Caliper Gauge after plate incubation at 30°C for 24 h. The minimal inhibitory concentration (MIC) of cefotaxime was tested on LB agar plates with an antibiotic concentration ranging from 30 to 0.0125 μg*mL^–1^.

The antibiotic treatment of *Arabidopsis* and rice seedlings was performed considering the MIC. Arabidopsis seeds (27-30 for each replicate) were surface sterilised and subsequently germinated in 1/2 Murashige Skoog 0.7% agar (MS, Duchefa Biochemie, Haarlem, Netherlands) medium plates containing 1.6 and 0.4 μg*mL^–1^ of cefotaxime. Seeds were vernalised for two days in the dark at 4°C, and then moved to a growing chamber (25°C, 12 h/12 h photoperiod, 56% humidity) for seven days. Seedling root length was measured using ImageJ2 (Rueden et al., 2017).

Rice seeds (7–10 for each replicate) were placed in sterile Magenta GA-7 vessels filled with sterile water containing 0.4 and 1.6 μg*mL^–1^ cefotaxime. The concentration was renewed every day for four days. The same antibiotic concentration was used during seed sterilization after dehulling, maintaining the seeds in antibiotic imbibition for 1 h.

To test the bacteria inoculum, Arborio, Lamone, Ermes and Arsenal rice seeds (6–10 for each replicate) were soaked with a fresh *Pantoea ananatis* bacterial suspension grown in LB medium until the OD_600_ = 0.5 and subsequently resuspended in PBS. Afterward, seeds were soaked for 1 h in the suspension and subsequent dilutions (Bertani et al., 2016), after de-hulling and standard surface sterilization without cefotaxime. Four-day rice coleoptiles were measured using a Lyon calliper gauge.

## Results

### Rice Seedling Bacterial Microbiota Under Air and Submergence

The bacteriome of Arborio and Lamone rice seedlings growing in submerged and aerobic conditions was identified to determine possible differences. The metagenomic analysis generated a total of 4,457,080 paired-end reads. After the quality check, one of the three replicates of Lamone rice varieties grown in aerobic conditions was removed, which then resulted in a total of 4,093,473 reads. After *in silico* depletion of chimeras and unassigned sequences, a total of 842 OTUs with 99% sequence similarity was detected (SILVA database).

**FIGURE 1:**
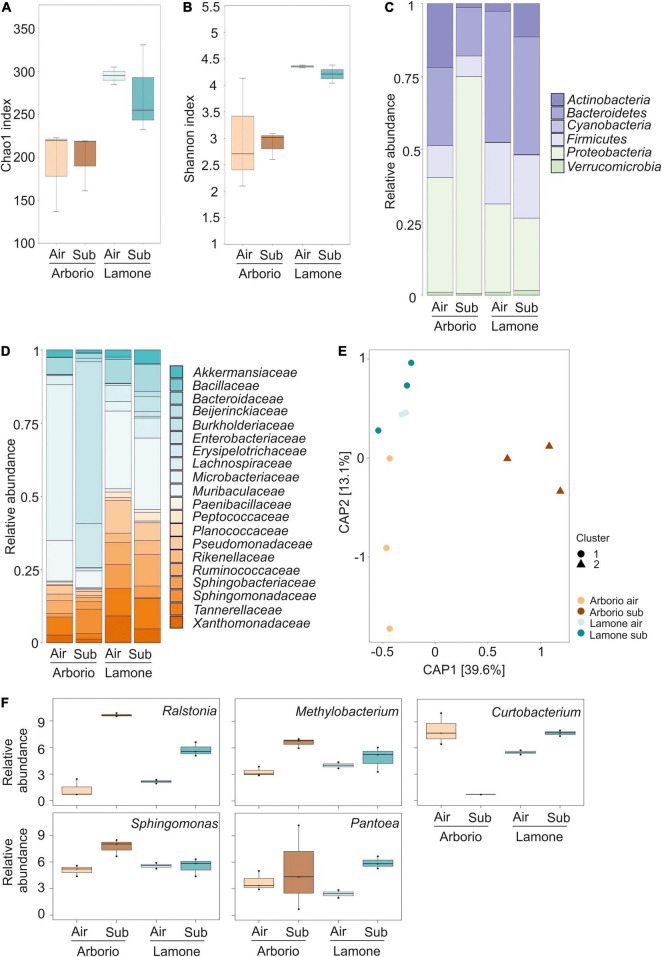
**(A)** Chao1 index reflecting the OTU abundance. Kruskal-Wallis test *p* = 0.055 **(B)** Shannon index reflecting the OTU diversity. Kruskal-Wallis test *p* = 0.073. **(C)** Relative abundance of the dominant phyla of Arborio and Lamone rice seedlings. **(D)** Relative abundance of the most abundant families of Arborio and Lamone rice seedlings. Only phyla and families showing a minimum average relative abundance of 20% are included in the figures, in which the average of the three replicates is represented. For Lamone air, two replicates are represented. **(E)** Canonical analysis of principal coordinates of the Bray-Curtis distance. **(F)** Relative abundance of the significant genus (FDR < 0.05) obtained grouping all the OTUs likely belonging to that single genus.

Alpha-diversity was estimated using the Chao1 and Shannon indexes, which revealed that the Arborio samples were less rich and diverse than the Lamone variety samples (Figures 1A,B), regardless of the growth conditions (Wilcoxon Rank Sum test Arborio *versus* Lamone, *P* < 0.005 for Chao1 index and *P* < 0.01 for Shannon index).

Taxonomic classification of the OTU phylum level identified *Bacteroidetes*, *Firmicutes* and *Proteobacteria* as the most abundant in the rice seedling bacterial community (Figure 1C). At the family taxonomic level (Figure 1D), *Bacteroidaceae* and *Microbacteriaceae* were well represented in the rice seedling microbiota.

Beta-diversity analysis was computed using both Bray-Curtis and weighted Unifrac distances which were visualised as a canonical analysis of principal components (CAP) with a constraint in the interaction between “variety” and “treatment” (Figure 1E and Supplementary Figure 1). Both matrixes showed a different microbiota composition of Arborio under submergence, grouping the three different replicates into a separate cluster from the other samples. The Bray-Curtis CAP was confirmed by the permutation analysis of variance (PERMANOVA), which indicated that 20.98% of the observed variation was attributed to the variety (*P* = 0.003), and that 19.17% was due the treatment (*P* = 0.006). The interaction between the variables explained 18.34% of the variation (Adonis test, 5000 permutations, *P* < 0.05). The interaction between treatment and variety variables was only significant (*P* = 0.014) in the Bray-Curtis analysis. The PERMANOVA analysis of weighted UniFrac CAP provided similar results.

The analysis of different OTUs available in rice seedlings and referred to different genus identified individual units that were differentially abundant among the rice samples. Some of the genera to which these OTUs belong, i.e., *Curtobacterium, Methylobacterium, Ralstonia, Sphingomonas* and *Pantoea*, were significantly different among varieties and growth conditions (FDR < 0.05) (Figure 1F).

MicFunPred tool was used to predict functional pathways abundance based on 16S rRNA datasets for significant Pfam terms among interesting groups (*p* < 0.001). A subgroup of 138 Pfam terms was identified and related to GO molecular functions. The GO molecular function showed a higher relative abundance in Arborio under submergence characterised by a separate clustering in comparison to the other samples (Supplementary Figure 2).

### Isolation and Identification of Culturable Microbial Strains

After 16S amplicon community sequencing, a bacterial culture collection of endophytes was generated. From the isolation procedures described in the Materials and Methods section, a collection of 42 putative endophytic bacteria, showing differences in colour and morphology, was isolated (Tables 1, 2, Bertani et al., 2016). The average number of culturable bacteria grown in aerobiosis was 2*10^3^ CFU*g^–1^ of rice seedlings. For rice grown under submerged conditions, the average was 7*10^6^ CFU*g^–1^ in air and 2*10^6^ CFU*g^–1^ under hypoxia.

**TABLE 1:** Putative bacterial endophytes isolated from Arborio and Lamone rice varieties at day 4 under dark air (Air) and submergence (Sub), and isolated under aerobic conditions.

Origin	Closest relative	Characteristic	Growth	MA
Lamone Air	*Bacillus altitudinis*	Endophytic (Potshangbam et al., 2018)		A
	*Bacillus firmus*	Endophytic (Pal et al., 2021)		A
	*Bacillus thuringiensis*	Endophytic (García-Suárez et al., 2021)		A
	*Brevundimonas mediterranea*	-		A
	*Kocuria rhizophila*	-		NA
	*Lysinibacillus parviboronicapiens*	-		A
	*Micrococcus luteus*	Endophytic (Vendan et al., 2010)		NA
	*Micrococcus yunnanensis*	Endophytic (Zhao et al., 2009)		NA
	*Paenibacillus rhizosphaerae*	Endophytic (Xia et al., 2013)		A
	*Paracoccus marinus*	-		A
	*Pseudomonas azotoformans*	Endophytic (Ma et al., 2017)		A
	*Sphingomonas desiccabilis*	Soil		A
Lamone Sub	*Microbacterium testaceum*	Endophytic (Zinniel et al., 2002)	FA	A
	*Pantoea eucalypti*	Endophytic (Wei et al., 2021)	FA	A
Arborio Air	*Bacillus firmus*	Endophytic (Pal et al., 2021)		A
	*Bacillus megaterium*	Endophytic (Bertani et al., 2016)		A
	*Bacillus niabensis*	-		A
	*Fictibacillus arsenicus*	-		NA
	*Fictibacillus phosphorivorans*	Endophytic (Khaskheli et al., 2020)		NA
	*Micrococcus yunnanensis*	Endophytic (Zhao et al., 2009)		NA
	*Nocardioides sp*			NA
	*Paenibacillus lactis*	Endophytic (Ahmad et al., 2019)		A
	*Paenibacillus rhizosphaerae*	Endophytic (Xia et al., 2013)		A
	*Pantoea eucalypti*	Endophytic (Wei et al., 2021)		A
	*Paracoccus marinus*	-		A
	*Rhodococcus cercidiphylli*	Endophytic (Li et al., 2008)		A
	*Rhodococcus yunnanensis*	-		A
Arborio Sub	*Bacillus megaterium*	Endophytic (Bertani et al., 2016)	FA	A
	*Pantoea eucalypti*	Endophytic (Wei et al., 2021)	FA	A
	*Pantoea ananatis*	Endophytic (Bertani et al., 2016)	FA	A
	*Xanthomonas sacchari*	Endophytic (Kumar et al., 2020)	FA	NA

*Taxonomic putative assignment was performed through 16S rRNA gene analysis using 27F and 1492R primers (Edwards et al., 1989) and querying SILVA and NCBI (similarity > 97%). Possible endophytic characteristic at the species level was taken from cited bibliography. Bacterial capacity of anaerobic respiration was directly investigated, testing the growth under hypoxia (Supplementary Figure 3). MA refers to genus availability (A) or non-availability (NA) in the metagenomics analysis, FA, facultative anaerobic.*

**TABLE 2:** Putative bacterial endophytes isolated from Arborio and Lamone rice varieties at day 4 under dark submergence (Sub), and isolated under hypoxia.

Origin	Closest relative	Characteristic	Growth	MA
Lamone Sub	*Kocuria rhizophila*	-	FA	NA
	*Microbacterium sp.*			A
	*Microbacterium testaceum*	Endophytic (Bertani et al., 2016)	FA	A
	*Paenibacillus sp.*			A
	*Pantoea eucalypti*	Endophytic (Wei et al., 2021)	FA	A
	*Pantoea vagans*	Endophytic (Bertani et al., 2016)	FA	A
Arborio Sub	*Pantoea vagans*	Endophytic (Bertani et al., 2016)	FA	A
	*Pantoea ananatis*	Endophytic (Bertani et al., 2016)	FA	A
	*Pantoea eucalypti*	Endophytic (Wei et al., 2021)	FA	A
	*Bacillus megaterium*	Endophytic (Bertani et al., 2016)	FA	A
	*Xanthomonas sacchari*	Endophytic (Kumar et al., 2020)	FA	NA

*Taxonomic putative assignment was performed through 16S rRNA gene analysis using 27F and 1492R primers (Edwards et al., 1989) and querying SILVA and NCBI databases (similarity > 97%). Possible endophytic characteristic at the species level was taken from cited bibliography. Bacterial capacity of anaerobic respiration was directly investigated, testing the growth under hypoxia (Supplementary Figure 3). MA refers to genus availability (A) or non-availability (NA) in the metagenomics analysis, FA, facultative anaerobic.*

Samples of Lamone and Arborio grown in the dark, in air and under submergence were used to identify aerobic bacteria (Table 1). Among the 31 strains isolated, 8 were not found in the above meta-analysis, which was likely due to the filters used for the OTU table generation after DNA sequencing. Many of these bacterial taxa were already found to be putative endophytes in previous analyses (Table 1). From the rice samples grown under submergence, only a few bacteria were isolated under aerobic conditions (Table 1). The majority of bacterial strains obtained in submerged rice samples and cultivated under aerobiosis, were found to be facultative anaerobic (Supplementary Figure 3).

The rice seedling samples obtained under dark submergence were used to identify culturable facultative anaerobic bacteria (Table 2). A total of 11 culturable bacteria were identified. Bacteria isolated under hypoxia were tested for their capacity to grow under both an aerobic and hypoxic state (Supplementary Figure 3), confirming that some have the capacity to grow under O_2_ shortage.

### Plant Growth Promotion of Isolated Bacteria

In the group of bacteria isolated from Arborio and Lamone samples grown in aerobic conditions, several species were found to have beneficial activities to plants, i.e., capacity to hydrolyse starch, produce indole-related compounds, solubilise phosphate, and to fix nitrogen (Table 3).

**TABLE 3:** *In vitro* activities of putative endophytic bacteria isolated from Arborio and Lamone rice varieties and reported in Tables 1, 2.

Activities in aerobic conditions					

Origin	Closest relative	IRC	SH	N_2_ fix	P sol
Lamone air	*Bacillus altitudinis*	−	−	+	−
	*Bacillus thuringiensis*	+	−	+	−
	*Brevundimonas mediterranea*	−	−	+	−
	*Kocuria rhizophila*	+	−	−	−
	*Micrococcus yunnanensis*	+	−	+	−
	*Pseudomonas azotoformans*	−	−	+	+
	*Sphingomonas desiccabilis*	−	−	+	+
Lamone sub	*Pantoea eucalypti*	+	−	+	+
Arborio air	*Bacillus firmus*	−	+	+	+
	*Bacillus megaterium*	−	−	+	+
	*Bacillus niabensis*	+	+	+	−
	*Fictibacillus arsenicus*	+	+	−	−
	*Fictibacillus phosphorivorans*	+	+	−	−
	*Micrococcus yunnanensis*	−	+	+	−
	*Paenibacillus lactis*	−	+	−	−
	*Pantoea eucalypti*	+	−	+	+
	*Rhodococcus cercidiphylli*	+	+	+	−
Arborio sub	*Bacillus megaterium*	+	−	+	+
	*Pantoea eucalypti*	+	+	+	+
	*Pantoea ananatis*	+	−	+	+
	*Xanthomonas sacchari*	−	+	−	−

**Activities under hypoxic conditions**			

**Origin**	**Closest relative**	**IRC**	**SH**

Lamone sub	*Paenibacillus sp.*	−	+
	*Pantoea eucalypti*	+	−
	*Pantoea vagans*	+	−
Arborio sub	*Pantoea eucalypti*	+	+
	*Pantoea ananatis*	+	−
	*Pantoea vagans*	+	−
	*Xanthomonas sacchari*	−	+

*IRC, indole-related compounds production; SH, starch hydrolysis; N_2_ fix, nitrogen fixation; P sol, phosphate solubilisation; +, detected; −, not detected.*

Some of the isolates identified under anaerobic conditions also showed PGP activities (Table 3). *Pantoea ananatis*, *Pantoea eucalypti* and *Pantoea vagans* related strains showed a certain capacity to produce indole related compounds, even if the intensity of the pink colour was lower in comparison to air, while *Paenibacillus sp, Pantoea eucalypti and Xanthomonas sacchari* related strains showed starch degradation activity.

### Bacteria Involvement in Rice Seedling Traits Under Submergence

**FIGURE 2:**
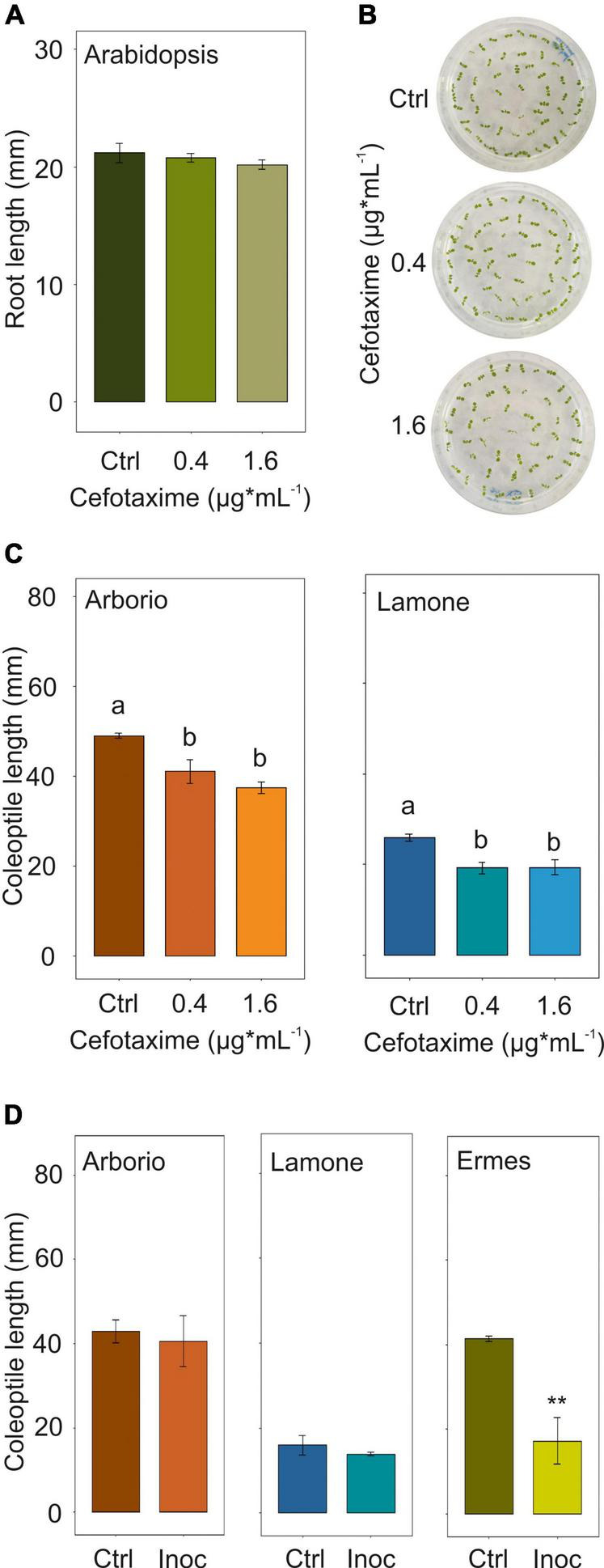
**(A)** Arabidopsis root growth up to 7 days after germination, using cefotaxime MIC 0.4 ug*mL^–1^ and a further concentration of 1.6 ug*mL^–1^ in the medium. Data are the mean ± SD (*n* = 3). ANOVA test. **(B)** Arabidopsis seedling phenotype at day 7 after germination using cefotaxime MIC 0.4 ug*mL^– 1^ and a further concentration of 1.6 ug*mL^– 1^ in the medium. **(C)** Lamone and Arborio coleoptile length at day 4 under dark submergence, adding cefotaxime MIC 0.4 ug*mL^– 1^ and a further concentration of 1.6 ug*mL^– 1^ in the medium. Data are the mean ± SD (*n* = 3). ANOVA test *p* < 0.05. Tuckey *post hoc* test **(D)** Lamone, Arborio and Ermes coleoptile length at day 4 under dark submergence, inoculating the seed with bacteria suspension. Data are the mean ± SD (*n* = 3). Student’s *t*-test, *p* ≤ 0.01.

Antibiotic assays were performed in order to test inhibition of isolated bacteria. The antibiotic susceptibility test, conducted on bacterial isolates identified in rice under submergence, identified cefotaxime as a possible controller of submergence-related bacteria (Table 4). The subsequent definition of the minimal inhibitory concentration (MIC) identified the concentration of 0.4 μg*mL^–1^ as effective (Supplementary Figure 4). The cefotaxime MIC and a further concentration of 1.6 μg*mL^–1^ was applied to *Arabidopsis* seedlings grown for 7 days on vertical plates to identify possible phenotype alterations. The treatment did not significantly affect the root length (Figure 2A) and neither were signs of stress evident on the leaves (Figure 2B).

**TABLE 4:** Antimicrobial susceptibility test applying *in vitro* diffusion on bacteria isolated from rice samples under submergence and showing PGP activities under hypoxia from Table 3.

	CTX	KZ	TE	K	CN	S	AMP	P
**Lamone**								
*Paenibacillus sp*	42 ± 2a	30 ± 1b	39 ± 2a	25 ± 1b	29 ± 2b	−	31 ± 1	36 ± 4
*P. eucalypti*	32 ± 1a	29 ± 3a	30 ± 2a	20 ± 1b	22 ± 1b	20 ± 1	23 ± 1	−
*P. vagans*	31 ± 1a	28 ± 1b	28 ± 1b	18 ± 1c	23 ± 1d	17 ± 1	22 ± 1	−
**Arborio**								
*P. eucalypti*	34 ± 3a	20 ± 2b	24 ± 3b	21 ± 2b	23 ± 1b	18 ± 3	14 ± 1	8 ± 1
*P. ananatis*	36 ± 2a	15 ± 2d	28 ± 2b	21 ± 1c	23 ± 1c	18 ± 2	13 ± 1	−
*P. vagans*	31 ± 6a	32 ± 1a	28 ± 1a	18 ± 1b	23 ± 3b	17 ± 1	23 ± 1	9 ± 1
*X. sacchari*	29 ± 1c	−	35 ± 1a	24 ± 1d	31 ± 1b	21 ± 1	25 ± 1	21 ± 1

*Results are the mean ± SD of the diameter (mm) of the inhibition from three individual colonies after applying the antibiotic disk containing kanamycin (K, 30 μg), streptomycin (S, 25 μg), ampicillin (AMP, 10 μg), cefotaxime (CTX, 30 μg), penicillin (P, 6 μg), tetracycline (TE, 30 μg), cephazolin (KZ, 30 μg), gentamicin (CN, 30 μg). For each species, the effect of different antibiotic disks content of 30 μg was compared (n = 3) (Two-way ANOVA test, p < 0.05, letters represent Tukey’s post hoc test).*

Arborio and Lamone varieties were germinated under submergence plus/minus cefotaxime addition in the medium. Interestingly, the cefotaxime MIC treatment showed a significant reduction in the coleoptile length in both varieties (Figure 2C). The same treatment was applied to seeds germinating in air, showing a similar final coleoptile length in both the varieties (Supplementary Figure 5).

Arborio and Lamone seeds were inoculated, after standard surface sterilisation, with the *P. ananatis* related strain, which was isolated in Arborio samples (long coleoptile) under submergence and was able to synthesise indole related compounds. The two rice varieties inoculated with the bacterial isolate did not show any significant variation in coleoptile length at day 4 under submergence in comparison to the uninoculated samples (Figure 2D).

In addition, two rice accessions characterised by different coleoptile length under submergence, Ermes (long) and Arsenal (short), were also inoculated with *P. ananatis* related strain at the same concentration than Arborio and Lamone and subsequent dilutions (Figure 2D and Supplementary Figure 6). Interestingly, Ermes showed significant differences in coleoptile length following bacterial inoculation (Figure 2D).

## Discussion

Endophytes active under low O_2_ may influence the first phase of seed germination, coleoptile elongation and thus seedling establishment under submergence. In order to select only endophytes available inside the seeds, thus those that are likely to be vertically transmitted, we removed the hull and sterilised the seed surface. Previous results showed that, in open air, the removal of endophytic bacteria from rice seeds dampens the development and increases vulnerability to pathogens (Verma et al., 2017), indicating the importance of this resource.

The bacteria microbiota composition of Arborio (long coleoptile) and Lamone (short coleoptile) varieties identified the prevalence of *Bacteroidetes*, *Firmicutes* and *Proteobacteria* (Figure 1C) that are well represented endophytic phyla detected in seeds of various plants (Truyens et al., 2015; Simonin et al., 2022). Seed microbiota generated by analysing several *Oryza* spp accessions has identified the prevalence of the phylum *Proteobacteria* and the abundance of *Bacteroidetes* and *Firmicutes* (Kim et al., 2020). *Proteobacteria* was also the most abundant phylum identified in wild and domesticated barley roots (Bulgarelli et al., 2015).

The bacteria community was unusual in the Arborio variety under submergence, which is characterised by a long coleoptile (Magneschi and Perata, 2009; Nghi et al., 2019). The bacterial families found only in Arborio grown under submergence included *Burkholderiaceae* (55%), that were predominant, and *Sphingomonadaceae* (9%) (Figure 1D). Notably, the presence of these families was barely detected in Arborio seedlings grown in air, suggesting an enrichment due to the submergence conditions. The *Burkholderiaceae* family is ubiquitous in diverse environments and is associated with several plant species (Carrión et al., 2018). This family was found to be abundant in *Oryza* wild and cultivated species (Kim et al., 2020).

The analysis of functional pathway abundance for significant Pfam terms among interesting groups showed an enrichment in Arborio under submergence (Supplementary Figure 2), confirming again the unique characteristic in comparison to Lamone and the samples in air. Indeed, microbial diversity of seeds is influenced by genotype, as recently shown in barley seeds (Bziuk et al., 2021).

Interestingly, *Methylobacterium*, *Sphingomonas* and *Ralstonia* genus were shown to be associated with an increase in abundance under submergence in both rice species (Figure 1F). *Sphingomonas* was previously found to be isolated more frequently in samples obtained from submerged rice plants (Bertani et al., 2016).

Only a few strains of bacteria were isolated from seedlings grown under dark submergence, confirming the stress imposed by this condition (Table 3). These includes *Pantoea*, *Bacillus*, *Microbacterium* and *Paenibacillus* genera that have been suggested to be part of the culturable core of plant endophytic bacteria isolated in seeds (Riva et al., 2022). *P. ananatis*, *P. eucalypti* and *P. vagans* related strains were found to produce indole-related compounds under hypoxia (Table 3). The metagenomics analysis confirmed the higher abundance of the genus *Pantoea* in both Lamone and Arborio varieties under submergence (Figure 1F). In previous reports, the analysis of a *japonica* rice diversity panel identified accessions with long and short coleoptiles (Nghi et al., 2019), in which the *AUXIN TRANSPORTER 1* (*AUX1*) plays a role in coleoptile elongation and thus seedling establishment during submergence (Nghi et al., 2021). This suggests that the production of auxin by bacteria may be an advantage for plants.

Plant growth promoting activities were tested in the putative endophytic bacteria identified both in air and submergence conditions. In air, twelve isolates belonging to Lamone and Arborio were positive to the production of indol-related compounds, sixteen showed nitrogen fixation ability and nine phosphate solubilisation capacity (Table 3). Interestingly, the capacity to degrade starch was identified only in strains isolated in Arborio variety, which displayed a longer coleoptile under submergence. However, under hypoxic conditions both Lamone and Arborio related isolates showed the capacity to degrade starch and produce indol-related compounds.

In order to reduce the bacteria community and assess the subsequent rice phenotype, we tested antibiotic sensitivity of strains isolated under submergence. *P. ananatis*, *P. eucalypti* and *P. vagans* related strains were found to be sensitive to cefotaxime MIC of 0.4 μg*mL^–1^ (Supplementary Figure 4). Cefotaxime has been widely used in plant tissue culture, since the low phytotoxic effect and the capacity to control bacteria contamination (Kaur et al., 2008; Asif et al., 2013; Naderi et al., 2016). In Arabidopsis and *japonica* rice varieties, the concentration of cefotaxime used for successfully plant tissue regeneration ranged between 100 and 500 μg*mL^–1^ (De Buck et al., 1998; Nishimura et al., 2007). Interestingly, the treatment of rice with this antibiotic showed a reduction in coleoptile length under submergence in both varieties harbouring a long and short coleoptile (Figure 2C). No effect were detected in Arabidopsis phenotype and in rice coleoptile under air, suggesting that bacteria sensitive to cefotaxime may be positively involved in the rice coleoptile length under submergence.

*Pantoea ananatis* is in the group of bacteria isolated in samples of Arborio seedlings grown under submergence, and this species has been previously isolated from rice plants grown under submergence conditions (Bertani et al., 2016). *P. ananatis* related strain was shown to have PGP activity under hypoxia, i.e., a capacity to produce indole-related compounds (Table 3). Several members of the *Pantoea* genus have been identified as plant pathogens (Walterson and Stavrinides, 2015) however, some isolates also possess PGP activities and can be used in agricultural applications (Megías et al., 2018; Suman et al., 2020; Lu et al., 2021). Different *P. ananatis* strains have diverse effects on maize seeds, ranging from weakly pathogenic to beneficial and growth-promoting effects (Sheibani-Tezerji et al., 2015). Interestingly, the pathogenesis of *P. ananatis* appears to be strain-specific (Kim et al., 2012; Doni et al., 2021). Our *P. ananatis* isolate did not show any pathogenic symptoms in rice seedlings and adult plants. The absence of genes for pathogenesis in *P. ananatis* may define its ubiquitous nature in rice genotypes (Raj et al., 2019; Kim et al., 2020) and identify strains for potential PGP application.

Plant inoculation studies with *P. ananatis* did not result in any variation in the coleoptile length in Arborio and Lamone varieties (Figure 2D). This could be due to the need for *P. ananatis* to interact with other endophytes in the microbiota which were not part of our experimental set up. Indeed, the richness of probiotic bacterial inoculant was shown to increase success of inoculation and improve plant growth (Hu et al., 2021). Alternatively, cefotaxime may indirectly influence the rice phenotype, and the coleoptile length may be the result of the plant genotype only, which can be dominant over the microbiome effect. The results obtained with Arabidopsis seedlings and rice coleoptile length in air, however, indicate that this may not be the case because of the absence of a phenotype following the treatment with cefotaxime.

We infected other rice varieties with *P. ananatis* related strain, i.e., Ermes and Arsenal, characterised by a long and a short coleoptile under submergence, respectively. The result showed a variation in coleoptile length of Ermes, also in accordance with the concentration of the inoculum applied (Figure 2D and Supplementary Figure 5). This indicates the possible role of *P. ananatis* on specific rice genotypes. However, we cannot exclude the presence of mechanisms in some rice accessions, which may reduce the capacity of certain bacteria to colonise the seed. A GWAS study on Arabidopsis accessions infected with *Pseudomonas simiae* showed genetic variations in the root and shoot traits after exposure to bacteria (Wintermans et al., 2016). This supports the hypothesis that the plant response to endophytic bacteria may vary depending on the plant genotype.

In summary, the result of this study is a first prove that endophytes, likely transmitted from the seeds to the offspring, are beneficial under low O_2_. We characterised for the first time the rice seedling bacterial community under submergence, showing a peculiar structure of the microbiota associated to the long coleoptile harbouring Arborio variety. We also proved that endophytic bacteria of rice seeds grown under O_2_ shortage show plant growth promoting activities that include starch degradation and indole-related compounds production. In fact, the capacity of rice to degrade starch under O_2_ deprivation and the contribute of auxin to coleoptile elongation are crucial for a successful seedling establishment and subsequent rice growth. Interestingly, the rice variety responds differently to bacteria inoculum, a characteristic that needs to be considered for possible practical applications. Using a selected microbial inoculum to burst growth under submergence would be very important for direct rice seeding practices.

## Data Availability Statement

The names of the repository/repositories and accession number(s) can be found below: https://www.ncbi.nlm.nih.gov/, PRJNA804691 and https://www.ncbi.nlm.nih.gov/genbank/, OM585508 to OM585549.

## Author Contributions

CP and PP conceived the work. CP designed and supervised the experiments and wrote the article, which was revised by all the authors. VV and IB managed the isolation of bacteria and PGP analysis, which were performed by GA. MD’A managed the meta-analysis, which was performed mainly by EG-Á with the help of GA. GA performed the experiments on plant materials. EG-Á and GA performed statistical analysis. All authors contributed to the article and approved the submitted version.

## Conflict of Interest

The authors declare that the research was conducted in the absence of any commercial or financial relationships that could be construed as a potential conflict of interest.

## Publisher’s Note

All claims expressed in this article are solely those of the authors and do not necessarily represent those of their affiliated organizations, or those of the publisher, the editors and the reviewers. Any product that may be evaluated in this article, or claim that may be made by its manufacturer, is not guaranteed or endorsed by the publisher.

## Footnotes

1
www.arb-silva.de/

2
https://www.ncbi.nlm.nih.gov/

3
www.eucast.org
