# Supplementary material for: Bacterial Endophytes Contribute to Rice Seedling Establishment Under Submergence
Source: Front Plant Sci. 2022 Jun 29;13:908349. doi: 10.3389/fpls.2022.908349 (PMC9277545; doi:10.3389/fpls.2022.908349)

Supplementary Material

Supplementary Figure 1. Permutational multivariate analysis of variance (PERMANOVA, Weighted-Unifrac distance).

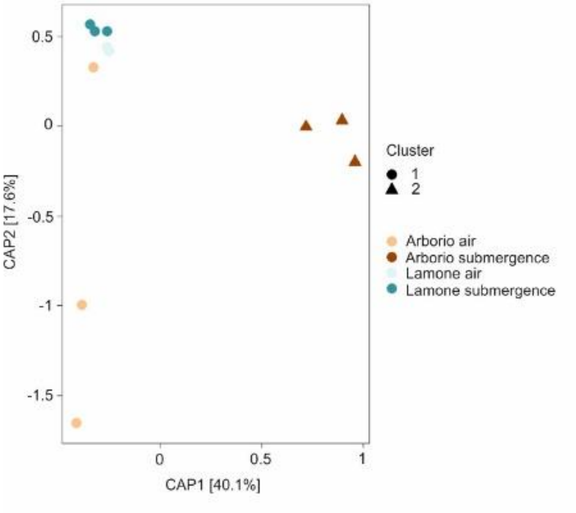

Supplementary Figure 2. Relative abundance of Gene Ontology (GO) molecular function of significant protein family (Pfam) prediction abundance based on MicFunPred analysis and summing the categories.

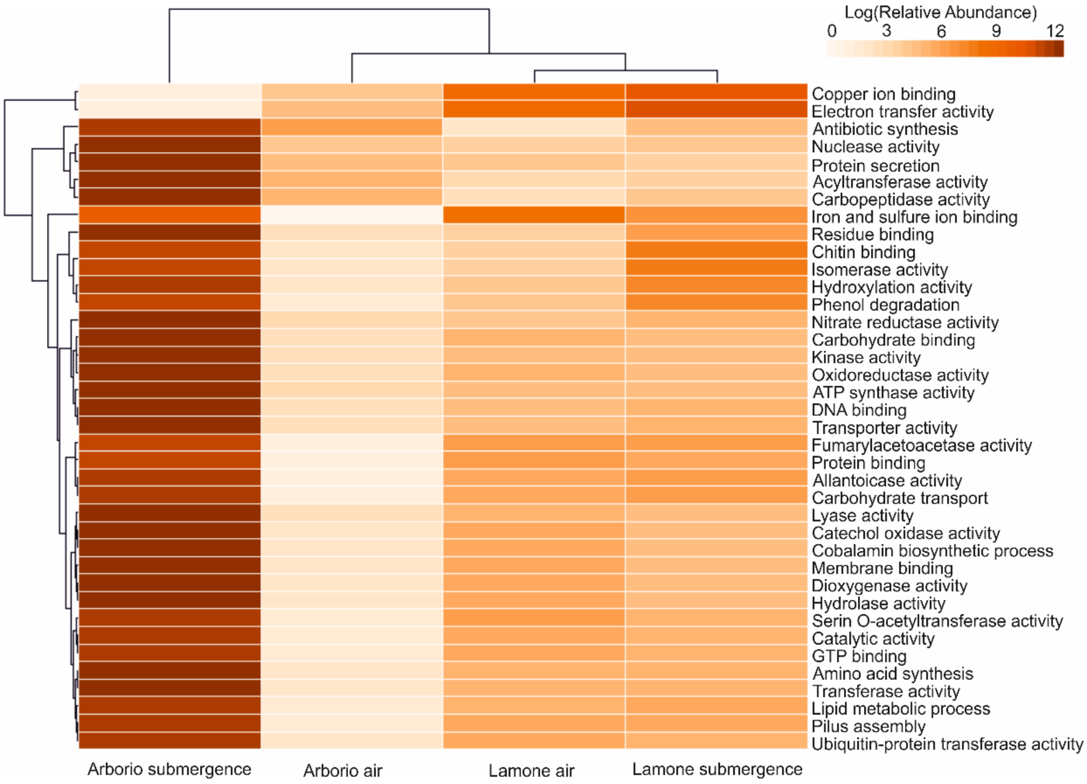

Supplementary Figure 3 - Capacity of aerobic and hypoxic growth of isolates identified in Arborio and Lamone under submergence from **Table 2**, scored through bacterial growth in air and hypoxic conditions. The analysis was repeated three times for each isolate with similar results.

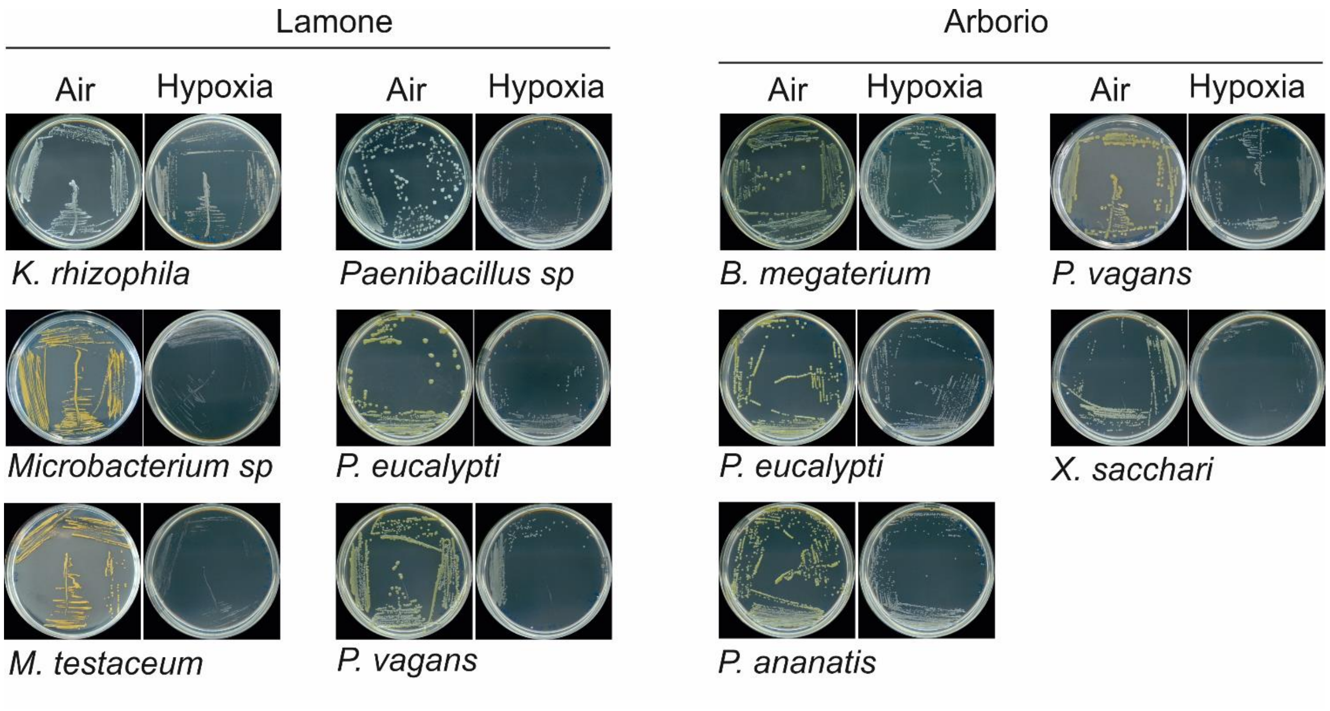

Supplementary Figure 4. Cefotaxime minimal inhibitory concentration (MIC) of isolates identified in Arborio and Lamone under submergence for the control of *Pantoea* spp growth *in vitro.*

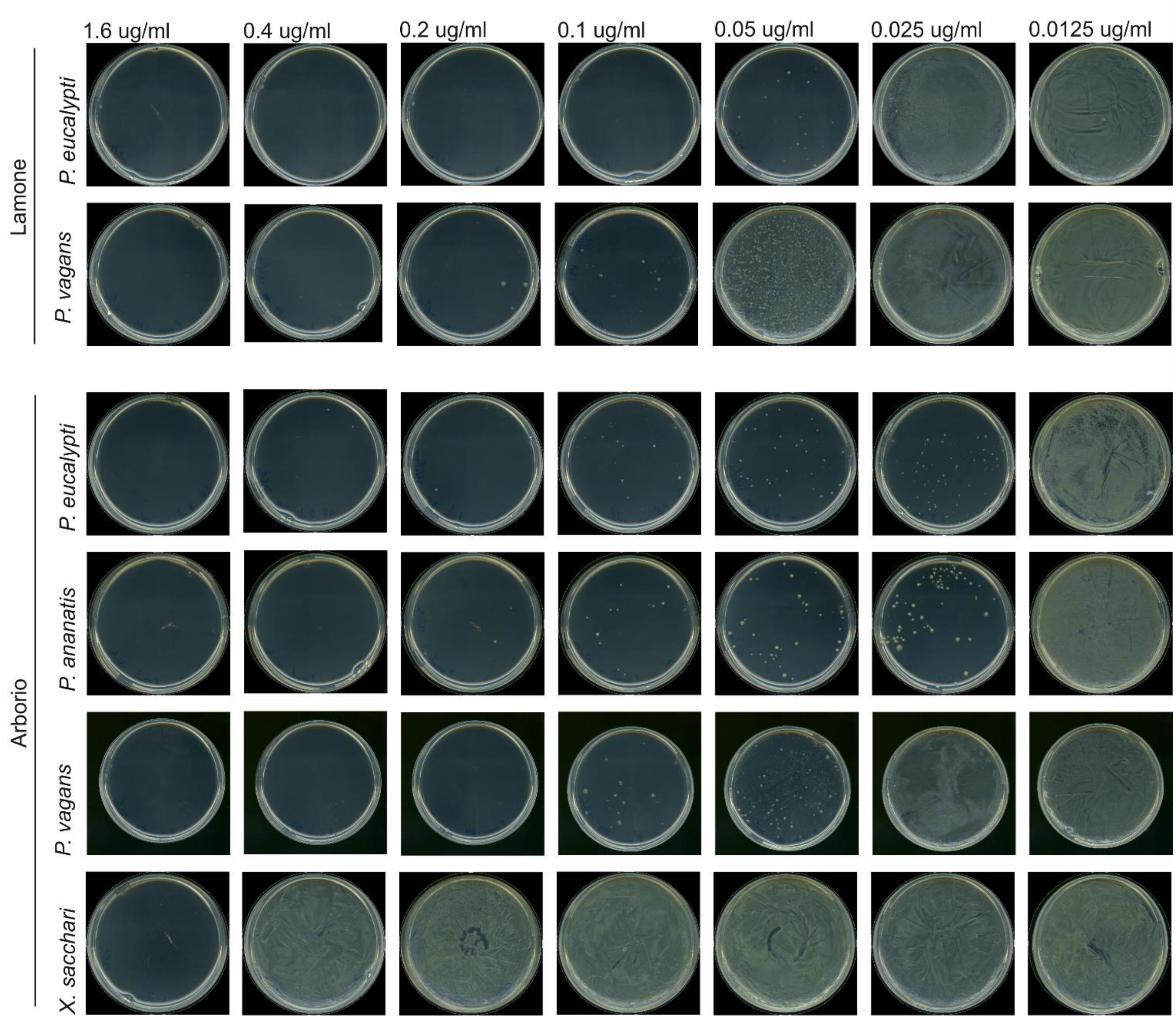

Supplementary Figure 5. Lamone and Arborio coleoptile length at day 4 under dark air, adding MIC 0.4 ug mL^{-1} in the medium. Data are the mean ± SD (n=3). Anova test $p<0.05$.

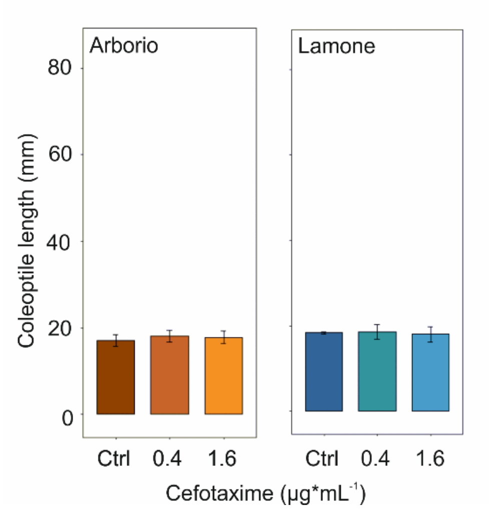

Supplementary Figure 6. Ermes (long) (information from **Figure 2D**) and Arsenal (short) coleoptile length with different bacteria concentrations. Data are the mean ± SD (n=3). ANOVA, $p \leq 0.005$.

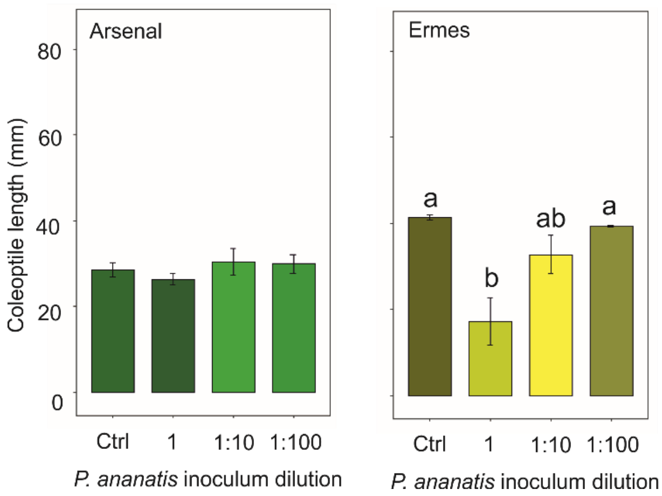

Supplement: Supplementary file 1 [file Data_Sheet_1.pdf]
